# Supplementary material for: A Global Screen for Assembly State Changes of the Mitotic Proteome by SEC-SWATH-MS
Source: Cell Syst. 2020 Feb 26;10(2):133–155.e6. doi: 10.1016/j.cels.2020.01.001 (PMC7042714; doi:10.1016/j.cels.2020.01.001)

Q9BVC3 | DCC1_HUMAN | DSCC1 DCC1 UNQ9337/PRO34008
Monomer MW [kDa]: 44.825 Monomer expected elution fraction: 47

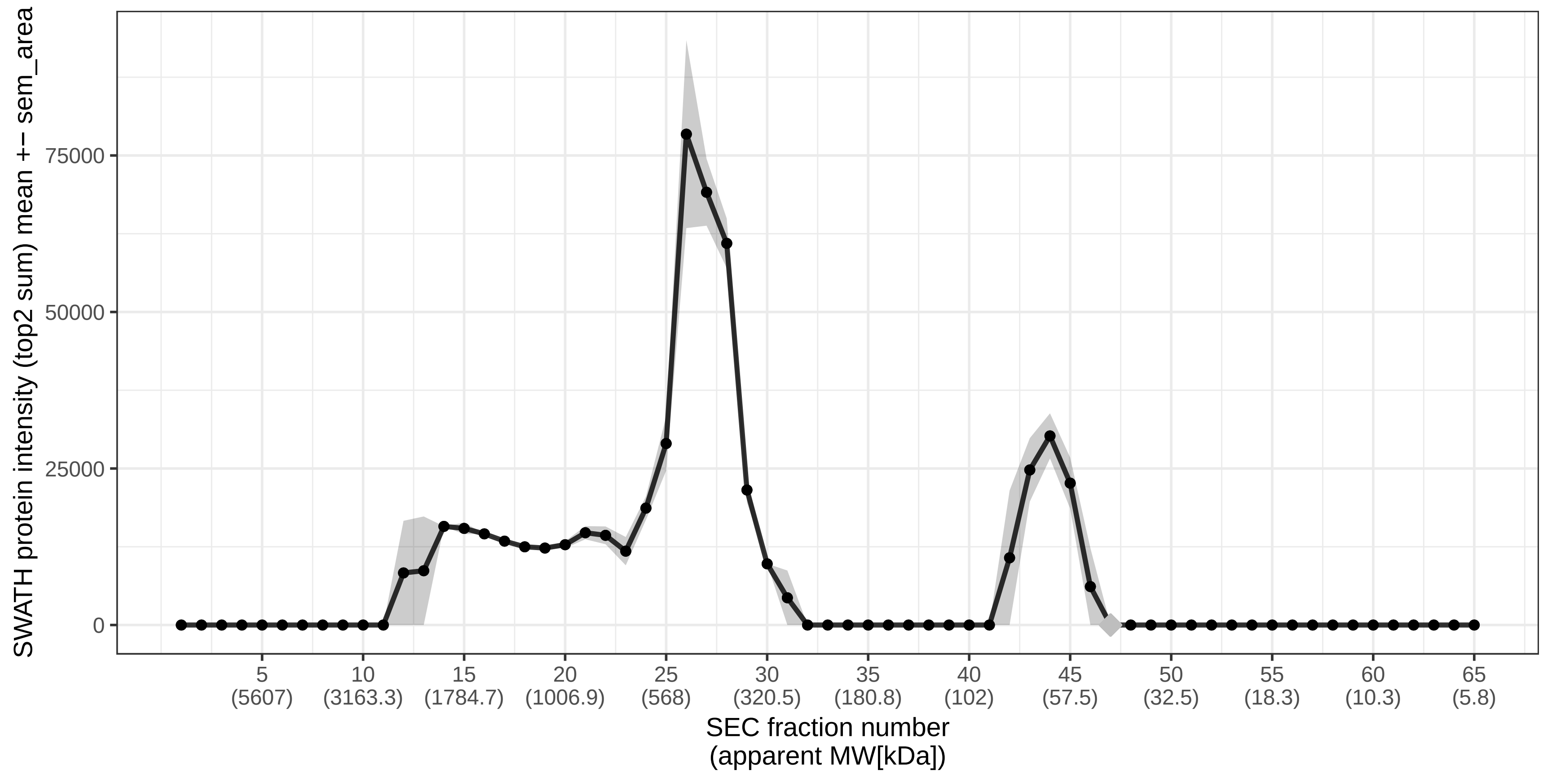

Supplement: Data S1. SEC-SWATH-MS Protein Chromatograms, Related to Figure 1 [file mmc6.zip › SECchrom_Q9BVC3_DCC1_HUMAN_DSCC1_DCC1_UNQ9337_PRO34008.pdf]
